# Cytidine-5-diphosphocholine supplement in early life induces stable increase in dendritic complexity of neurons in the somatosensory cortex of adult rats

**DOI:** 10.1016/j.neuroscience.2008.04.017

**Published:** 2008-08-13

**Authors:** V. Rema, K.K. Bali, R. Ramachandra, M. Chugh, Z. Darokhan, R. Chaudhary

**Affiliations:** National Brain Research Centre, NH-8, Nainwal Mode, Manesar, Haryana-122050, India

**Keywords:** citicholine, barrel cortex, Golgi–Cox, stroke, plasticity, Sholl analysis, CDP-choline, cytidine-5-diphosphocholine, DAPI, 4′,6-diamidino-2-phenylindole, DMEM, Dulbecco's minimal essential medium, MAP2, microtubule-associated protein 2, P, postnatal day

## Abstract

Cytidine-5-diphosphocholine (CDP-choline or citicholine) is an essential molecule that is required for biosynthesis of cell membranes. In adult humans it is used as a memory-enhancing drug for treatment of age-related dementia and cerebrovascular conditions. However the effect of CDP-choline on perinatal brain is not known. We administered CDP-choline to Long Evans rats each day from conception (maternal ingestion) to postnatal day 60 (P60). Pyramidal neurons from supragranular layers 2/3, granular layer 4 and infragranular layer 5 of somatosensory cortex were examined with Golgi–Cox staining at P240. CDP-choline treatment significantly increased length and branch points of apical and basal dendrites. Sholl analysis shows that the complexity of apical and basal dendrites of neurons is maximal in layers 2/3 and layer 5. In layer 4 significant increases were seen in basilar dendritic arborization. CDP-choline did not increase the number of primary basal dendrites on neurons in the somatosensory cortex. Primary cultures from somatosensory cortex were treated with CDP-choline to test its effect on neuronal survival. CDP-choline treatment neither enhanced the survival of neurons in culture nor increased the number of neurites. However significant increases in neurite length, branch points and total area occupied by the neurons were observed. We conclude that exogenous supplementation of CDP-choline during development causes stable changes in neuronal morphology. Significant increase in dendritic growth and branching of pyramidal neurons from the somatosensory cortex resulted in enlarging the surface area occupied by the neurons which we speculate will augment processing of sensory information.

Cytidine-5-diphosphocholine (CDP-choline) is being used as a neuroprotective and memory enhancing drug in conditions such as aging (Alvarez et al., 1997; Teather and Wurtman, 2003) and other neurovascular diseases (see review by Adibhatla and Hatcher, 2005). Its role as a crucial intermediate in the biosynthesis of cell membrane phospholipids is well established (Kennedy and Weiss, 1956). Orally administered CDP-choline is hydrolyzed to choline and cytidine (Agut et al., 1993) and subsequently taken up from the blood by brain and other tissues (Lopez-Coviella et al., 1995). In the brain choline gets metabolized to phosphorylcholine (Millington and Wurtman, 1982) which then combines with CTP to generate intracellular CDP-choline which is subsequently converted to phosphatidylcholine (Adibhatla et al., 2004). Choline in the brain also gets converted to acetylcholine especially in cholinergic neurons (Blusztajn and Wurtman, 1983). If there is reduction in acetylcholine levels in the brain then choline from neuronal membrane phosphatidylcholine is used for the synthesis of acetylcholine (Maire and Wurtman, 1985; Ulus et al., 1989).

Injuries such as middle cerebral artery occlusion have been shown to cause reduction in phosphatidylcholine (Adibhatla et al., 2006). Since phosphatidylcholine is an integral component of cell membranes any change in its concentration would affect membrane stability and cause damage to the cell which could result in neurological deficits. In an experimental model of stroke in adult rats Hurtado et al. (2007) have seen increase in branch orders and spine numbers on basilar dendrites of layer V pyramidal neurons from motor cortex following chronic CDP-choline treatment. Although stroke is known to occur during pregnancy (Mas and Lamy, 1998; Sloan and Stern, 2003; Jaigobin and Silver, 2000) and at perinatal ages (Nelson, 2007) the effect of treatment with CDP-choline on neuronal structure of the developing brain is not known.

We therefore addressed two questions in this study (1) Does exogenously supplemented CDP-choline during the developmental period of the brain produce changes in dendritic arborization of neurons? (2) Are these changes stable? We administered CDP-choline throughout gestation up to postnatal day (P) 60. This time period was chosen because it encompassed the temporal changes in NMDAR1 expression that occurs in the somatosensory cortex from early development until attaining adult levels by P60 (Rema and Ebner, 1996). Our results show that CDP-choline treatment increases the complexity of dendritic arborization and this effect is sustained long term. The consequence of this enhanced dendritic complexity potentially increases the total synaptic contact area and could facilitate processing within a neural network.

## Experimental procedures

All experimental procedures carried out for this study were approved by the NBRC Institutional Animal Ethical Care Committee and followed NIH guidelines. Long Evans rats used for this study were on 12-h light/dark cycle with free access to food and water and housed in temperature-controlled room (25 °C). In each cage one female rat and one male rat were placed for mating. To ensure that there was no social isolation the females were separated from their mates only during last week of gestation. The pups were weaned from their mother on P21 and two pups of the same sex were housed in each cage until they were adults. The number of animals used for this study was the minimum required, and care was taken to minimize their suffering.

### CDP-choline administration

Experimental group consisted of pups born to female animals that were administered CDP-choline (Sigma-Aldrich, St. Louis, MO, USA) at a concentration of 100 mg/kg orally once a day in the morning. We used this concentration based on the studies by Agut et al. (1984) which showed that a dose of 100 mg/kg body weight of CDP-choline administrated orally was neuroprotective in rats. A stock solution of the drug was made in water each day. From the first day of gestation the pregnant dams were weighed daily and the required amount of CDP-choline solution applied on two pieces of cornflakes was fed to the rats. After birth from P1 up to P14 (P0 is day of birth) CDP-choline was administered to the pups in liquid form. The pups were weighed and the required amount of CDP-choline solution was aliquoted directly into the mouth using a micropipette. From P14 till P60 CDP-choline was given to the pups with cornflakes as described above. Control group consisted of animals treated in similar manner as experimental animals except that water was used instead of CDP-choline. Morphological analyses of neurons from somatosensory cortex were done 6 months later by Golgi–Cox staining of neurons.

### Golgi–Cox staining

Brains from 11 male rats aged P240 which included five controls and six CDP-choline-treated (one from each litter) were used for Golgi–Cox staining. Animals were deeply anesthetized and perfused transcardially with normal saline (0.9% NaCl). The brains were removed and cut into three blocks and immersed in Golgi–Cox solution (Glaser and van der Loos, 1981) for 14 days in the dark during which time the Golgi–Cox solution was changed every 2 days. The brains were then sunk in 30% sucrose solution, sectioned at 200 μm thickness on a vibratome and processed for Golgi–Cox staining as described by Gibb and Kolb (1998).

### Rat primary cortical cultures

Cortical cultures were established from somatosensory cortex of normal P0 Long Evans pups following modified procedure of Palmer et al. (1999). The tissue was minced and incubated in a solution containing papain (2.5 U/ml, Sigma-Aldrich) and DNAse (250 U, Sigma-Aldrich) for 5 min at 37 °C and cells were dissociated by gentle pipetting in Dulbecco's minimal essential medium (DMEM). After washing the cells three times in DMEM they were suspended in plating medium containing DMEM, l-glutamine, B27, N2 and penicillin/streptomycin 100 U/ml (all media components were from Gibco, Invitrogen Corporation, Carlsbad, CA, USA). Cells were plated onto polylysine-d-lysine (Sigma-Aldrich) and laminin (Sigma-Aldrich) precoated glass coverslips at a density of 10^5^ cells per coverslip in 12 well Nunc plate and cultured at 37 °C and 5% CO_2_. At 6 h following plating (after the cells had attached to the substrate) the medium was replaced with fresh medium. CDP-choline (Sigma-Aldrich) treatment began from the second day in culture. For the treatment the medium was changed and replaced with medium containing 50 μM or 100 μM CDP-choline every alternate day. Control cultures were not treated with CDP-choline. The cultures were maintained for 15 days and then fixed with 4% paraformaldehyde. Four sets of cultures (each set consisting of one control, one 50 μM CDP-choline-treated and one 100 μM CDP-choline-treated coverslip) were prepared for one experiment. The experiment was repeated thrice. The cultures were immunoreacted with antibodies to microtubule-associated protein 2 (MAP2) to visualize the neurons using the method described in Rema and Ebner (1999). The coverslips were mounted on slides with mounting media containing 4′,6-diamidino-2-phenylindole (DAPI) (Vector Laboratories, Burlingame, CA, USA) to stain all the nuclei.

### Neuronal tracing and data analysis

Pyramidal neurons from the layers 2/3, layer 4 and layer 5 of somatosensory cortex with complete cellular and dendritic staining were selected for analysis. These neurons had a prominent apical dendrite pointing toward the pial surface. Digital reconstruction of the neurons was done in 3-D using Neurolucida (MicroBrightField, Colchester, VT, USA) and Microfire camera attached to a Nikon Eclipse E800 microscope. We used Golgi–Cox staining technique because with this technique visualization of more neurons from all layers of the cortex was easily possible.

The morphological parameters measured for each neuron were (i) total apical dendritic length, (ii) total basilar dendritic length, (iii) branch order, (iv) total number of branch points (nodes), and (v) total area occupied by the cell body and its dendrites determined using convex hull analysis. These features of neurons were determined from the reconstructed neuronal data and quantitative analysis was done using Neuroexplorer software (MicroBrightField). The complexity of dendritic arborization was obtained from Sholl analysis by calculating the number of dendritic branches that intersect defined concentric circles spaced 25 μm apart starting from the center of cell body as a function of distance from soma.

### Neuron and total cell counts

The effect of CDP-choline treatment on neuronal and total cell survival was determined from the primary cultures of somatosensory cortex. Neurolucida software was used to count the cells from the serial images that were taken of the entire culture area from each condition. For determining the total cell counts DAPI-stained nuclei were counted. The values obtained for control condition was taken as 100% and used as reference to determine changes in the experimental conditions. For estimating the number of neurons, MAP2-labeled cells were counted to calculate the percent of neurons with respect to the total cells in each coverslip. Neuronal morphology of cultured neurons was determined by tracing and analyzing five neurons, from each dish, using Neurolucida software. From each condition 40 neurons were traced and analyzed using the methods similar to those used for the *in vivo* experiments as described above.

### Statistical analysis

From each animal three to five neurons were traced from each layer. The values of all neurons from each layer were averaged for each individual animal. This mean value was taken as the unit of analysis. Statistical tests were performed using repeated measures analyses of variance (ANOVA) to compare experimental and control groups. The level of significance between control and experimental conditions was taken at *P*≤0.05. All statistical tests were performed using SigmaStat.

## Results

Animals were weighed every day for the initial 2 months and twice a week for the rest of the experiment. Their behavior in standard home cages was also visually monitored. We did not find differences in the weight or behavior in their home cages between the control group and the CDP-choline administered group.

**Fig. 1:**
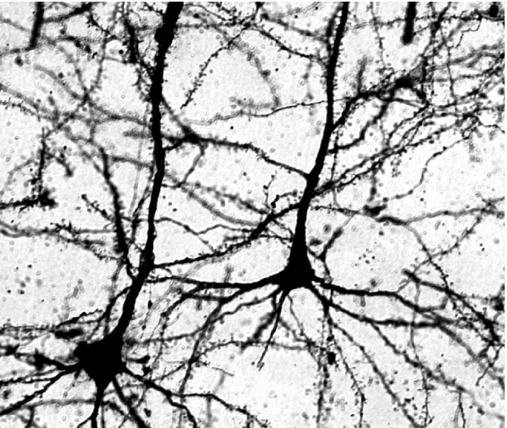
Photomicrograph of pyramidal neurons from somatosensory cortex of a CDP-choline-treated rat showing complete impregnation of cell body and dendrites stained with the Golgi–Cox method.

In this study we used Golgi–Cox staining technique to visualize neurons from layers 2/3, layer 4 and layer 5 of somatosensory cortex of CDP-choline-treated and control rats brains because this technique robustly labeled a limited number of neurons throughout the cortex. Fig. 1 shows photomicrograph of the Golgi–Cox stained pyramidal neurons from the somatosensory cortex of a CDP-choline-treated rat. Neurons with complete impregnation of cell body and dendrites which were unobstructed by other cells and dendrites were selected for tracing and reconstruction of the entire dendritic tree. All analyzed neurons from all layers had a prominent apical dendrite which projected toward the pial surface.

**Fig. 2:**
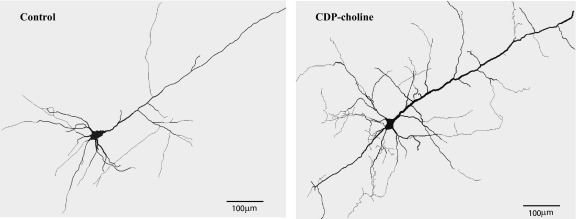
Neurolucida tracings of pyramidal neurons from somatosensory cortex of control and CDP-choline administered rats. Note the increase in dendritic arborization after CDP-choline treatment (scale bar=100μm).

To determine the effect of CDP-choline on the dendritic morphology we traced the neurons from CDP-choline-treated animals and controls (Fig. 2). We compared complexity in arborization of apical dendrites and basal dendrites for neurons from each layer.

### Effect of CDP-choline on apical dendritic morphology

**Fig. 3:**
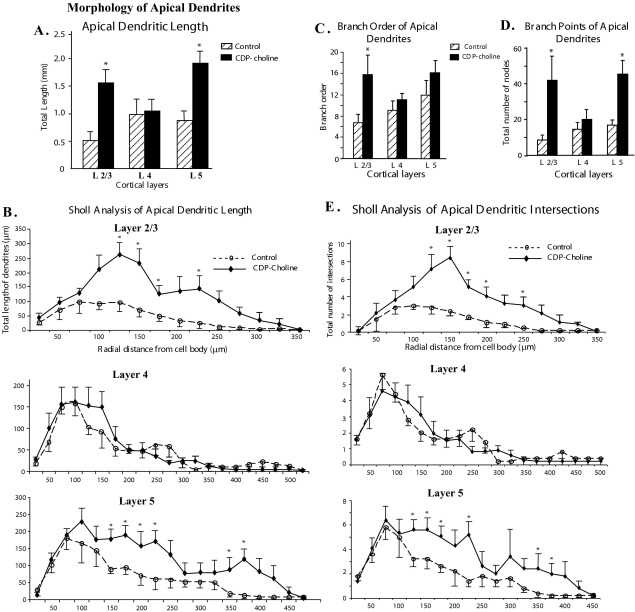
Effect of CDP-choline on apical dendritic morphology. (A) Histogram showing the total length of apical dendrites for neurons in layers 2/3, 4 and 5. CDP-choline administration significantly increased sum total length of apical dendrites (which includes the total length of primary dendrite and all apical dendritic branches) of neurons in layers 2/3 and 5 of the somatosensory cortex. (B) Line graph illustrating the total length of apical dendrites of neurons from layers 2/3, 4 and 5 in 25 μm concentric rings, from the cell body to the distal ends of dendrites, using Sholl ring analysis. Note the increase in length mainly in the middle section of the apical dendrites of neurons in layer 2/3 and 5 from somatosensory cortex of CDP-choline-treated rats. (C) Branch order analyses of apical dendrites for neurons from different cortical layers. CDP-choline treatment significantly increases the branches order on neurons in layers 2/3. (D) Branch point analysis of apical dendrites for neurons from layers 2/3, 4 and 5. The significant increase in number of nodes (branch points) in layers 2/3 and 5 suggests complex branching pattern in higher order branches in CDP-choline-treated animals. (E) Line graph showing the total number of intersections of apical dendrites in 25 μm concentric rings, from cell body to distal ends of the dendrites, using Sholl ring analysis of neurons from different layers. Note the increase in apical dendritic intersections of neurons in layers 2/3 and 5 indicating higher complexity in branching pattern as compared with neurons in layer 4.

Comparison of arborization of apical dendrites of neurons in the somatosensory cortex between CDP-choline-treated and control animals is shown in Fig. 3. The total apical dendritic length (which included the length of the primary apical dendrite and the length of all apical dendritic branches) in CDP-choline-treated rats was significantly more in neurons within layers 2/3 (*P*=0.006) and layer 5 (*P*=0.02) (Fig. 3A). However apical dendritic morphology of layer 4 neurons was not affected by CDP-choline treatment. The complexity of apical dendritic arborization was determined by Sholl analysis by measuring the total dendritic length and number of dendritic intersections within each concentric shell of 25 μm radial distances extending from the cell body to the distal end of dendrites. Upon examination of the dendrites from proximal to distal regions we saw significant increase in the total length of dendrites in the middle regions of the dendrites of neurons from layers 2/3 and layer 5 in CDP-choline-treated animals (Fig. 3B). However the apical dendrites of layer 2/3 neurons in CDP-treated animals were more complex as seen by the significant increase in branch order (Fig. 3C). The total number of branching points in the apical dendritic tree (Fig. 3D) is significantly more in layers 2/3 (*P*=0.04) and layer 5 (*P*=0.007). Sholl analysis also reflected a similar increase in the number of intersections of dendritic branches (Fig. 3E) in layers 2/3 and 5.

### Effect of CDP-choline on basal dendritic morphology

**Fig. 4:**
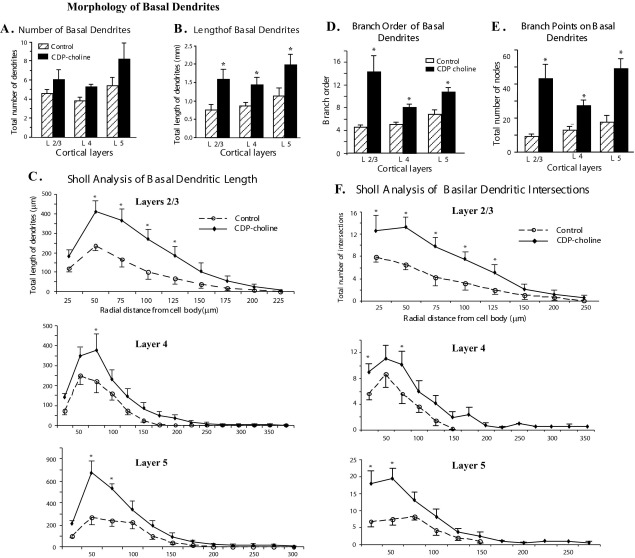
Effect of CDP-choline on basal dendritic morphology. (A) Histogram showing total number of basal dendrites of neurons in layers 2/3, 4 and 5 of somatosensory cortex. CDP-choline administration does not increase the number of primary basal dendrites of neurons from layers 2/3, 4 and 5 of somatosensory cortex. (B) Total length of basal dendrites of neurons in layers 2/3, 4 and 5 of somatosensory cortex. Note the significant increase in total length of basal dendrites of neurons in layers 2/3, 4 and 5 of somatosensory cortex in CDP-choline-treated animals. (C) Sholl ring analysis of the length of basal dendrites of neurons in layers 2/3, 4 and 5 of somatosensory cortex from CDP-choline-treated and control rats. Note the significant increase in dendritic length of neurons is in the proximal 50 μm to 125 μm region from cell body. (D) Branch order analysis of basal dendrites for neurons in different layers of somatosensory cortex. Note the increase in the branch order on the neurons from layers 2/3, 4 and 5 in CDP-choline-treated rats. (E) Total number of branch point on basal dendrites of neurons in different layers of somatosensory cortex. CDP-choline administration increases number of branch points in neurons from layers 2/3, 4 and 5 of somatosensory cortex. (F) Line graph showing the total number of intersections of basal dendrites in 25 μm concentric rings, from cell body to distal ends of dendrites, using Sholl ring analysis of neurons from somatosensory cortex. Neurons of layers 2/3, 4 and 5 from CDP-choline-treated rats show higher dendritic intersections in proximal concentric shells.

Analyses of basal dendritic morphology was done using the same methods as those used to analyze the apical dendritic tree (Fig. 4). CDP-choline administration does not increase the number of primary basal dendrites (Fig. 4A) but the total length of the basal dendrites is significantly enhanced in neurons from layers 2/3 (*P*=0.02), layer 4 (*P*=0.03) and layer 5 (*P*=0.04) as shown in Fig. 4B. Further examination of dendritic length by Sholl analysis revealed that the total length of dendritic branches in each successive 25 μm concentric ring around the cell body throughout the entire dendritic tree is more in neurons of CDP-choline-treated animals (Fig. 4C). This increase was significant in 50 μm to 125 μm radius from cell body of neurons from layers 2/3 and in 50 μm to 75 μm radius from cell body of neurons from layer 4 and layer 5 of CDP-choline-treated animals.

We estimated the complexity of branching by using both branch order analysis and by measuring the number of branch points. As shown in Fig. 4D, administration of CDP-choline significantly increased the of branch order on basilar dendrites of neurons in the three layers within the somatosensory cortex (*P*=0.007 for layer 2/3; *P*=0.002 for layer 4 and *P*=0.005 for layer V). Increase in the number of branch points on the basilar dendritic trees was also observed in CDP-choline-treated animals (*P*=0.005 for layer 2/3; *P*=0.005 for layer 4; *P*=0.002 for layer 5). As an additional measure of complexity we examined the dendritic intersections with Sholl analysis in 25 μm radial distances extending from the cell body to distal ends of basilar dendrites as shown in Fig. 4F. CDP-choline treatment increases the number of intersections of dendritic branches of all basal dendrites of neurons in all layers along the entire dendritic tree.

#### Effect of CDP-choline on dendritic field

**Fig. 5:**
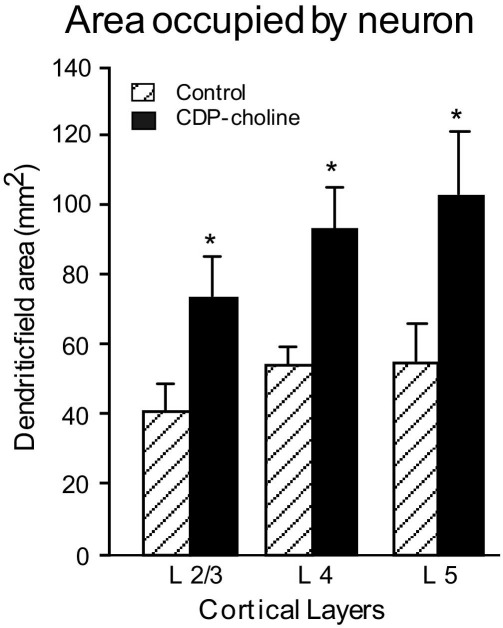
Convex hull analysis of neurons from layers 2/3, 4 and 5 somatosensory cortex of control and CDP-choline administered rats. CDP-choline treatment increases the total dendritic field of neurons in layers 2/3, 4 and 5 of somatosensory cortex resulting in an increase in the area occupied by the neurons.

Measurement of the total area occupied by the neuron was done using convex hull analysis. As shown in Fig. 5 CDP-choline treatment increased the overall area occupied by the neurons in supragranular layers 2/3 (*P*=0.043), granular layer 4 (*P*=0.046) and infragranular layer 5 (*P*=0.053) of the somatosensory cortex indicating that the neurons in these layers have larger dendritic fields than controls.

#### Effect of CDP-choline on cell survival

**Fig. 6:**
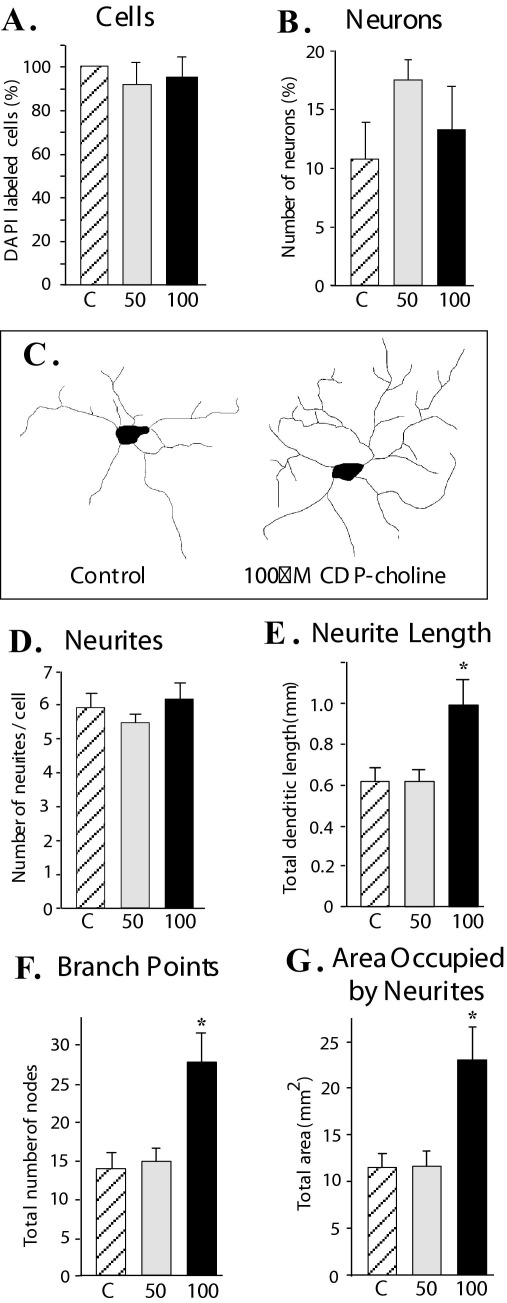
Effect of CDP-choline on survival and morphology of neurons in culture. CDP-choline treatment (50 μM or 100 μM) of primary cultures from somatosensory cortex does not increase survival of (A) the total number of cells, and (B) the total number of neurons. See Experimental Procedures section for details of analysis. (C) Neurolucida tracing of neurons from control and CDP-choline-treated cultures. (D) The number of primary neurites arising from the neurons is not higher in the CDP-choline-treated condition compared with the control cultures (which similar to *in vivo* results as shown in Fig. 4A). At higher concentration of CDP-choline (100 μM) there is significant increase (E) in total length of neurites, (F) number of branch points and (G) area occupied by neurites. For all histograms hatched bar (labeled C) depicts data from control cultures, gray bar (labeled 50) depicts data from cultures treated with 50 μM CDP-choline and black bar (labeled 100) depicts data from cultures treated with 100 μM CDP-choline.

Primary cultures generated from the somatosensory cortex of normal Long Evans P0 pups were treated with CDP-choline (50 μM or 100 μM) to determine its effect on survival of cortical cells. To estimate the total number of cells in each condition the cultures were labeled with the nuclear stain DAPI and the total number of neurons was determined by labeling the neurons with MAP2. CDP-choline treatment did not have any effect on the total number of cells (Fig. 6A) nor did it increase the number of neurons in both the experimental groups as compared with the controls (Fig. 6B). Morphological details of traced neurons from the cultures (Fig. 6C) were determined and analyzed using same parameters as were used for *in vivo* analysis described above. The average number of neurites per neuron was also not different among all three groups (Fig. 6D). However similar to *in vivo* results, neurons in cultures treated with 100 μM CDP-choline showed significantly longer neurites (Fig. 6E) with more branch points (Fig. 6F) and occupied a larger area (Fig. 6G).

## Discussion

We found that a daily dose of oral CDP-choline at 100 mg/kg/day during early development was sufficient to produce more dendritic branch points and longer dendrites on cortical neurons. These changes occurred at doses shown to increase dopamine release and dopamine dependent behavior in rats (Agut et al., 1984). Klein et al. (1990) have shown that administration of CDP-choline at a dose of 100 mg/kg/day increases the plasma choline concentration to 17 μM, which is above the concentration (14 μM) required to bring about net influx of choline into the brain. This increase in choline levels could facilitate synthesis of membrane phosphatidylcholine and provide sufficient choline to up-regulate the synthesis of acetylcholine thereby preventing degradation of membrane phosphatidylcholine (Adibhatla and Hatcher, 2005). These metabolic changes could be one of the possible mechanisms for the reduction in neurologic deficits seen in CDP-choline-treated stroke patients. Stroke also occurs during pregnancy (Mas and Lamy, 1998; Sloan and Stern, 2003; Jaigobin and Silver, 2000) and in young children (Nelson, 2007) and on the basis of known beneficial effects CDP-choline could emerge as a treatment for these patients. However the effect on developing neurons following administration of CDP-choline either during pregnancy or at early perinatal ages is unknown.

One of the goals of this study was to determine if CDP-choline administration during development of the cerebral cortex affects neuronal morphology. Analyses of NMDAR1 expression in the developing rat somatosensory cortex (Rema and Ebner, 1996) showed layer specific modulation of NMDAR1 up to 60 days postnatal until the stable adult state is achieved. Hence we administered CDP-choline during gestation through 2 months postnatal. The second goal of this investigation was to determine if the changes induced by CDP-choline administration were stable. Long-term pharmacological intervention should be avoided if it is possible to achieve stable beneficial effect in a shorter time interval of drug administration. No study has examined the stability of morphological changes in neurons following CDP-choline treatment. In this study we therefore examined whether the effect of CDP-choline supplement was stable over a relatively long time interval by analyzing the morphological changes after 6 months following CDP-choline administration.

Our results show that CDP-choline administration through gestation up to P60 enhances the dendritic morphology of neurons in the somatosensory cortex for a very long period after discontinuing the drug. We analyzed the dendritic length and branch points on the dendrites, total number of basal dendrites and area occupied by the dendritic arbors of neurons from all layers of the somatosensory cortex. Two major conclusions emerge from these studies: (i) CDP-choline administration during development increases the surface area occupied by neurons in the somatosensory cortex probably throughout life. There is no reason to believe that these changes are restricted to the area of cortex that we analyzed. (ii) The alterations in the neuronal morphology were detected after a period of 6 months following CDP-choline administration therefore we predict that these changes are likely to be stable and perhaps will persist even in aged rats. Such stability of morphological changes could be the likely mechanism for improvement in memory in 24 month old rats seen by Crespo et al. (2004) following oral administration of 150 mg CDP-choline per day for 12 months.

In a recent study Hurtado et al. (2007) observed that the improvement of motor behavior in animals with unilateral MCA occlusion following treatment with CDP-choline is accompanied by significant increase in number of branches as well as spines on basal dendrites of pyramidal neurons from layer 5 of motor cortex. However others have shown that cortical injuries induce dendritic growth of layer 5 neurons in the contralateral hemisphere (Jones and Schallert, 1994; Voorhies and Jones, 2002; Luke et al., 2004) and increase the number of spines (Adkins et al., 2002). It is therefore possible that the increase in dendritic branching and spines seen by Hurtado et al. (2007) in rats with MCA occlusion following i.p. injections of 1000 mg/kg CDP-choline treatment is influenced by the plastic changes resulting from injury to the brain in addition to the effect of CDP-choline on neuronal structure. Administering large doses of drug daily through the i.p. route over a long recovery period in humans might not be the best method for long-term treatment. We therefore tested whether a lower concentration of CDP-choline given by oral route for relative short time is capable of producing stable changes in neuronal morphology. In our study we found that 100 mg/kg of CDP-choline given as oral supplement was sufficient to induce stable morphological changes in both apical and basal dendrites of neurons in all layers of somatosensory cortex. However it is possible that a higher concentration is needed to produce similar changes after injury or in the adult brain.

Clinical trials have been carried out to test the effectiveness of citicholine (CDP-choline) administration in patients with acute ischemic stroke using doses ranging from 500 mg to 2000 mg per day with varying results on the effectiveness of the drug (Cohen et al., 2003; Clark et al., 1999; Dávalos et al., 2002). The high dose of 2000 mg/day, compared with 500 mg/day, was shown to produce some degree of recovery of function in about 30% of patients (Dávalos et al., 2002). The dosage of 100 mg/kg we have used in this study depends on the weight of the rat and therefore is on the order of approximately two to four times higher than the dose of 2000 g/day given to a human subject. These results suggest that the degree of benefit may depend in some way on the concentration of citicholine.

In the rat somatosensory cortex each layer is specialized in its input and output characteristics. We therefore did layer-wise classification of our data and found that CDP-choline administration influences dendritic arborization of pyramidal neurons in a layer specific manner. Neurons in layers 2/3 and 5 showed significant changes in most morphological parameters analyzed. These layer specific influences could have important implications for information processing within the somatosensory system. Neurons in layer 4 receive the major portion of sensory inputs from the lemniscal pathway through the thalamus and in turn project to neurons of layers 2/3 (Kim and Ebner, 1999; Petersen and Sakmann, 2001; Douglas and Martin, 2004; Shepherd and Svoboda, 2005). So it is possible that the sensory information that arrives in the cortex is processed in a similar manner in both control and CDP-choline-treated animals. But examination of the neuronal architecture in the thalamic sensory nuclei is needed to determine if there are differences that could modify the neuronal activity of layer 4. However it is likely that there might be mechanisms preventing large-scale morphological changes in layer 4. Hints of layer 4 neurons' stability are also seen in other studies. For example sensory deprivation during early postnatal development does not affect experience-dependent plasticity in layer 4 neurons of adult rats whereas the neurons in layers 2/3 show significant deficits (Rema et al., 2003). Also layer 4 neurons show temporal delay in achieving experience-dependent plasticity (Diamond et al., 1994).

Administration of CDP-choline during early development could influence the survival of neurons and glia. Studies that have examined the effect of CDP-choline on survival of neurons in disease conditions show contrasting results. In a Parkinson's disease model Barrachina et al. (2003) showed attenuation of substantia nigra (SN) dopaminergic cell dropout suggesting that CDP-choline could influence cell survival. CDP-choline at 100 μM concentration has been shown to reduce cell death in an *in vitro* model of ischemia (Hurtado et al., 2005). However Sato et al. (1988) did not observe a protective effect of CDP-choline on cell survival in brains of rats with forebrain ischemia. Our results indicate that CDP-choline does not affect proliferation or survival of cells in culture (Fig. 6). However we saw an increase in both branching and length of neurites in cultures treated with 100 μM concentration of CDP-choline. This observation is similar to our *in vivo* results. Thus, these results indicate that one of the main effects of CDP-choline is to increase dendritic branching around the neuron which in effect will increase the overall connectivity of neuron. It is possible that the effect of CDP-choline on the survival of neurons/cells in the brain is different from that seen in the cultures.

A key determinant of neuronal connectivity is dendritic morphology. Neurons of layers 2/3 have extensive intrahemispheric and interhemispheric cortical connections. They send axon collaterals to layer 5 of the same barrel column and to several adjacent barrels. They also have trajectories of projections to ipsilateral second somatosensory cortex, dysgranular cortex and to contralateral barrel cortex (Akers and Killackey, 1978; Olavarria et al., 1984; Chapin et al., 1987; Koralek et al., 1990; Fabri and Burton, 1991; Kim and Ebner, 1999). Apical dendrites of layer 5 pyramidal neurons project to layer 1 with branches that ramify in layers 2/3 and 4 (Schubert et al., 2001). Neurons in layer 5 are also known to project to thalamic, pontine and brainstem sensory nuclei. Several studies show that plasticity changes are more prominent in layers 2/3. In rats robust changes in experience-dependent plasticity were shown to occur first in layers 2/3 and in layer 5 (Diamond et al., 1994). Restricted neonatal sensory deprivation reduced both responsiveness to sensory stimulation and experience-dependent plasticity in neurons of layers 2/3 (Rema et al., 2003) and disrupts receptive field structure (Shepherd et al., 2003). Integration of sensory information through layer V neurons of the barrel cortex and cerebellum has been shown to be essential for whisker-guided sensory behavior (Jenkinson and Glickstein, 2000). Therefore our results showing a highly significant effect of CDP-choline on dendritic field arborization of neurons in layers 2/3 and 5 indicate that there is profound influence on information processing which would bring about fast recruitment of neurons within the network.
